# Clinico-Demographic Profile and Factors Affecting Duration of Hospital Stay Among Burn Patients in an Emergency Department of a Tertiary Care Center, South India: A Hospital-Based Cross-Sectional Study

**DOI:** 10.7759/cureus.43397

**Published:** 2023-08-12

**Authors:** Sasikumar Mahalingam, Gunaseelan Rajendran, Sathish Rajaa, Arshiya Aazmi, Nanda Maroju, Suruthi Purushothaman, Mounika Gara, Shivani Karn, Ajithkumar Rajendran, Vishwanath Balassoundaram

**Affiliations:** 1 Emergency Medicine, Aarupadai Veedu Medical College and Hospital, Puducherry, IND; 2 Community Medicine, Employees State Insurance Postgraduate Institute of Medical Sciences and Research, Chennai, IND; 3 Emergency Medicine, Karpaga Vinayaga Institute of Medical Sciences and Research, Chengalpattu, IND; 4 Surgery, Jawaharlal Institute of Postgraduate Medical Education and Research, Puducherry, IND; 5 Dermatology, Jawaharlal Institute of Postgraduate Medical Education and Research, Puducherry, IND; 6 Emergency Medicine, Jawaharlal Institute of Postgraduate Medical Education and Research, Puducherry, IND; 7 Emergency Medicine, Sri Ramaswamy Memorial (SRM) Medical College and Hospital, Trichy, IND

**Keywords:** tertiary care hospital, clinical profile, socio-demographic profile, duration of hospital stay, burns

## Abstract

Background

Burns continue to be a serious public health problem in India. It persists as an endemic disease in spite of implementing various preventive measures at the individual and community levels. Etiology and factors influencing burns are varied. There is a paucity of data regarding the clinico-demographic profile of burns disease, especially from emergency tertiary care settings in India.

Objective

To assess the proportion of burn patients having longer hospital stays (>1 week) and the influence of clinico-demographic factors associated with it among the burn patients presenting to the emergency department of a tertiary care institute in south India.

Methodology

An institution-based cross-sectional analytical study was conducted among burns patients attending the Emergency Medicine Department (EMD) of a tertiary care center between January 2017 and December 2017. Information on clinico-demographic profile and duration of hospital stay were captured using semi-structured data collection proforma.

Results

All the 327 burns injury patients who presented to our EMD during the study period were included. Among the 327 patients, 259 (79%) were admitted to the EMD. Among 259 admitted patients, 142 (55%) patients were discharged home. Among these 142 patients, 106 (74.6%; 95%CI 66.8-81.2) had longer hospital stays (more than one week). Female gender and facial/inhalational burns were found to have an independent effect on the length of hospital stay even after adjusted analysis.

Conclusion

Length of hospital stay is independently influenced by female gender and facial/inhalational burns. This study also identified the need for better home safety, child-proofing, proper pre-hospital care, and dedicated burns units in the community.

## Introduction

Burn injuries occur universally, beyond geographical boundaries, and adversely affect mankind. Injuries and deaths because of burns are mostly preventable. According to a 2018 World Health Organization report, every year, about three lakh people die from burns worldwide, whereas 1,80,000 people die from burn injuries, with the majority living in low- and middle-income countries [1]. Not just posing a burden to the health care system, burn injuries can even have an altogether different dimension as they can lead to varied social, psychological, and financial consequences not just among the affected persons but also among their family members [2].

The epidemiology of burns varies across the globe due to differences in literacy, civilization, social, and cultural activities. India being a country of diverse cultures and customs, clinical and epidemiological studies are required to evaluate the factors determining burns incidence. Burns have always remained a serious health problem in India, constituting more than seven million new incident cases, around twenty thousand burns-associated deaths, and more than one million nonfatal moderate-to-severe burns every year [1]. Deaths due to burns are among the leading cause of death in India. Among these injuries, 10% are severely life-threatening warranting admission and approximately 25% of cases getting admitted succumb to significant morbidity and mortality [3]. Nearly 2.8 lakh people require reconstructive surgeries and long-term rehabilitation therapy. In India, burn incidence is still a rising trend; whereas, among developed countries, burn incidence is reducing [3]. Injuries due to burns also pose severe health concerns due to the paucity of dedicated burn units, specialized doctors, lack of awareness among the public, and increased cost of treatment.

The etiologic factors associated with burns are different. The most common etiological factors are accidental, suicidal, or homicidal causes which may manifest as a spectrum ranging from mild burns to severe life-threatening injuries. The patient-specific risk factors, such as time taken to reach any point of care, severity, mechanism, and type of burns, primarily affect the outcome of the patient. This is of utmost importance, especially in developing countries like India, where the majority of the people are illiterate, live below the poverty line, and still rely on old methods of making food like stoves and chulhas. Thus, they are always surrounded by an unsafe environment. Burns patients can clinically present in different forms such as mild-to-severe burns, airway burns, circulatory shock, sepsis, acute respiratory distress syndrome, and cyanide or carbon monoxide poisoning with or without associated trauma. With regards to the duration of hospital stay, many factors pinch in such as the severity of burns, complications of burns, and total body surface area (TBSA) involved. Contrary to the belief, a longer duration of hospital stay has a better outcome because salvageable patients require better long-term care. 

Despite the huge burden imposed by burn injuries in day-to-day health care services, research to venture into the social and clinical profile of burn patients attending the Emergency Medicine Department (EMD) is still in its infancy in India. Such research will contribute to the baseline data for devising several community and hospital-level interventions. It will also facilitate the allocation of resources at the community level, with special emphasis on pre-hospital care. Therefore, this study was planned to set up a database of clinico-demographic profiles of patients with burns injuries attending a tertiary care hospital along with the main focus on length of hospital stay and factors determining it.

## Materials and methods

Study design, population, and setting

We conducted a hospital-based cross-sectional prospective analytical study in the EMD of a tertiary care center, in Puducherry, India, between January 2017 and December 2017. The study was conducted among the patients who presented with burns injury to EMD. Around 10,000 patients attend the EMD of the tertiary care center every month, of which 30-40 patients had burn injuries. All burn patients were initially resuscitated and treated in our EMD, and those who require admission were referred to the designated burns unit in the super-specialty department. Treatment options included resuscitation, escharotomy, grafting procedures, and reconstructive surgeries, which were available around the clock at our center.

Sample size

The sample size was calculated by OpenEpi v 3.01 with reference to a study done by Nadkarni et al. in 2017, taking the proportion of burns patients having a duration of hospital stay of more than one week to be 59.8% and a relative precision of 10%, confidence interval (CI) of 95%, and the sample size was calculated to be 257 [4]. However, we consecutively covered all the patients (N=327) who presented to the EMD with burns during the study period.

Data variables, study procedure, and data collection

The principal investigator (PI), posted at the EMD to provide emergency care, collected data from the patients attending the EMD. The patients who attended outside the PI’s shift hours were interviewed by trained medical doctors after giving them adequate training. The purpose of the study and the procedure involved in the study were explained to patients/relatives before getting consent. We captured the basic socio-demographic information like age, education, occupation, monthly family income, and place of injury using a pretested semi-structured questionnaire (refer to appendices (Figure 2)). The clinical profile of patients was recorded from the admission sheets and records maintained at the EMD. American burns association (ABA) severity grading system was used to classify the severity of burns. The outcome variable (length of hospital stay) was obtained from the hospital records during discharge and was categorized as less than one week and more than one week.

Ethical approval

The study protocol was reviewed and approved by Institutional Ethics Committee for observational studies of JIPMER (JIP/IEC/2016/1010). The purpose of the study was explained to the patients/relatives, and informed consent was obtained from them before the interview. Privacy and confidentiality were ensured.

Statistical analysis

Data was entered in Microsoft Excel (Microsoft Corporation, Redmond, USA) and analysis was done using SPSS version 20.0 (SPSS Inc, Chicago, IL, USA). Continuous variables such as age and length of hospital stay were summarized as mean and standard deviation (SD) or median and interquartile range (IQR) based on the Kolmogorov-Smirnov normality test. Categorical variables such as gender, place of injury, pre-hospital measures, and occupation were summarized as frequency and proportions. The outcome variable was then categorized as less than seven days and more than seven days. The association of categorical variables with the outcome variable was carried out by chi-square test/Fischer exact test, and an unadjusted prevalence ratio with a 95% CI was calculated.

Multivariable regression analysis (log-binomial model) was done to identify the independent factors determining the outcome of the study by taking variables with p-value <0.2 into the model, and adjusted prevalence ratios with 95% CI were calculated. A p-value of less than 0.05 was considered statistically significant. Simple linear regression analysis was also performed to obtain clinico-demographic predictors of the length of hospital stay among admitted patients. 

## Results

A total of 327 burn patients who attended the tertiary care center during the study period, consented, thereby, were included in the study. Among them, 152 (46.5%) belonged to the age group of 20-64 years, with equal representation of both genders. About 177 (54.1%) study participants were employed, and home (90%) was the most common location of the incident (Table 1).

**Table 1:** Socio-demographic characteristics of study participants who attended the EMD of a tertiary care center N=327 EMD, Emergency Medicine Department

Socio-demographic characteristics	N (%)
Age (in years)	
≤12	114(34.9)
13-19	40(12.2)
20-64	152(46.5)
≥65	21(6.4)
Gender	
Male	168(51.0)
Female	159(49.0)
Occupation	
Unemployed	24(7.4)
Employed	177(54.1)
Children, students	126(38.5)
Burns incident place	
Home	294(90.0)
Others (workplace, temple, school, playground)	33(10.0)

With respect to the clinical characteristics, thermal burns (94%) were the most common mechanism of injury, with accidental burns (74%) being the most commonly encountered mode of injury. Hot water spillage accounted for 40% of accidental burns, followed by accidental injury by cooking and stove bursts. The majority, 215 (66%), of the study participants had severe burns, while 196 (60%) had a delay of more than one hour to reach the first point of care. Around 123 (38%) had TBSA of 10-30%, and 28% had both facial and inhalational burns, whereas 48% were free of both. Traditional and sometimes inappropriate pre-hospital measures were provided for 35% of cases (Table 2).

**Table 2:** Clinical characteristics of study participants who attended the EMD of a tertiary care center N=327 II s, second degree superficial; II d, second degree deep; EMD, Emergency Medicine Department; ABA, American Burns Association; TBSA, total body surface area Severity of burns classification - refer to appendices (Table 6)

Clinical characteristics	N (%)
Mechanism of burns	
Thermal	307(94.0)
Electrical	11(3.3)
Chemical	9(2.7)
Mode of burns	
Accidental	241(74.0)
Suicidal	80(24.0)
Homicidal	6(2.0)
Degree of burns	
I	7(2.0)
II s	215(66.0)
II d	57(17.0)
III	48(15.0)
Severity of burns (ABA)	
Mild	73(22.0)
Moderate	29(12.0)
Severe	215(66.0)
First point of care delay	
≤1 hour	131(40.0)
>1 hour	196(60.0)
Burns TBSA %	
<10	70(21.4)
10-30	123(37.6)
31-50	56(17.1)
>50	78(23.9)
Pre-hospital measures	
Yes	115(35.2)
No	212(64.8)
Facial/inhalational burns	
Facial or inhalational	80(24.0)
Both	90(28.0)
None	157(48.0)

**Figure 1:**
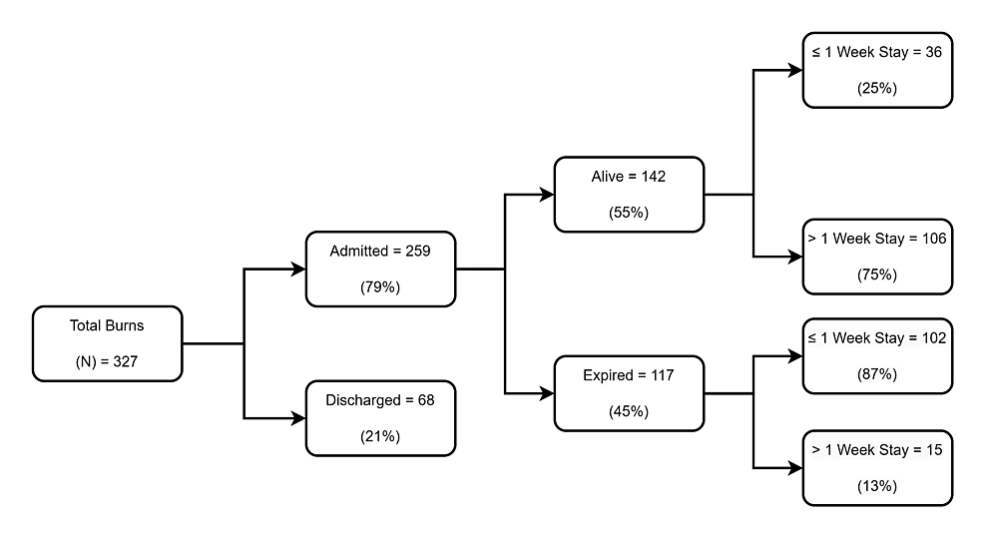
Flowchart depicting the admission and mortality status of the study participants

**Figure 2:**
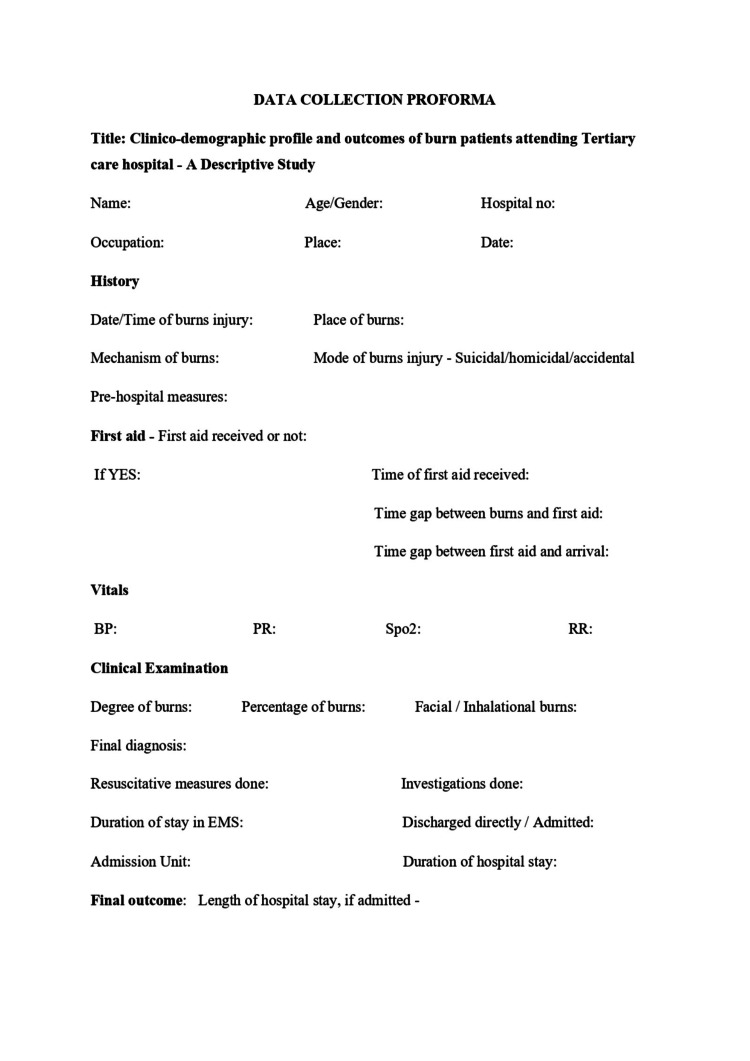
Burns data collection proforma

Of the 327 burn injury patients, 259 (79%) were admitted, and 68 (21%) were discharged directly from EMD owing to minor burns. Among those admitted, only 142 (55%) patients were alive, while 117 (45%) expired. Causes of death include septic shock, acute respiratory distress syndrome, and hypovolemic shock. The health center being a tertiary care institute, the majority of the caseload constituted referral cases from other health centers seeking tertiary care. Thus, as the expected majority, 102 (74%) of the admitted cases, expired within the first seven days. Since our primary objective was to determine the proportion of burns patients having a longer hospital stay (>1 week) and the factors associated with it, in order to obtain unbiased estimates of the factors influencing hospital stay among a homogenous group, we decided to run the analysis only among the alive 142 patients (Figure 1).

The proportion of study participants who had a longer duration of stay was 74.6% (95% CI 66.8-81.2), with a mean duration of 15.5 (12.4) days. Factors like female gender (prevalence ratio: 1.20 95% CI 1.00-1.44; p=0.04) and patients with both facial and inhalational burns (prevalence ratio: 1.35 95% CI 1.12-1.63; p<0.01) were found to be significantly associated with a longer hospital stay. The association between clinico-demographic characteristics with the duration of hospital stay is shown in Table 3.

**Table 3:** Clinico-demographic determinants of duration of hospital stay among admitted alive study participants in EMD of a tertiary care center N=142 PR, prevalence ratio; CI, confidence interval; EMD, Emergency Medicine Department; TBSA, total body surface area; ABA, American Burns Association Severity of burns classification - refer to appendices (Table 6)

Variable	≤1 week	>1 week	Total	Unadjusted PR (95% CI)	P-value
Gender					
Male	27(30.68)	61(69.32)	88	1(ref)	
Female	9(16.67)	45(83.33)	54	1.20(1.00-1.44)	0.049
Total	36(25.35)	106(74.65)	142		
Age group (years)					
≤12	19(30.65)	43(69.35)	62	1(ref)	
13-19	4(25.00)	12(75.00)	16	1.08(0.78-1.50)	0.640
20-64	9(16.98)	44(83.02)	53	1.19(0.97-1.47)	0.086
≥65	4(36.36)	7(63.64)	11	0.92(0.57-1.48)	0.723
Total	36(25.35)	106(74.65)	142		
Place of incident					
Home	33(23.98)	94(74.02)	127	1(ref)	
Others	3(20.00)	12(80.00)	15	1.08(0.82-1.42)	0.577
Total	36(25.35)	106(74.65)	142		
Burns TBSA (%)					
<10	5(45.45)	6(54.55)	11	1(ref)	
10-30	27(26.47	75(73.53)	102	1.35(0.78-2.34)	0.289
31-50	4(13.79)	25(86.21)	29	1.58(0.90-2.76)	0.108
Total	36(25.35)	106(74.65)	142		
Mechanism of burns					
Thermal	30(23.8)	96(76.19)	126	1.52(0.76-3.06)	0.238
Electrical	4(50.00)	4(50.00)	8	1(ref)	
Chemical	2(25.00)	6(75.00)	8	1.5(0.67-3.34)	0.321
Total	36(25.35)	106(74.65)	142		
Mode of burns					
Suicidal	0(0)	11(100)	11	-	
Homicidal	1(50.00)	1(50.00)	2	1(ref)	
Accidental	35(27.13)	94(72.87)	129	1.46(0.36-5.85)	0.595
Total	36(25.35)	106(74.65)	142		
Degree of burns					
II s	26(25.49)	76(74.51)	102	1(ref)	
II d	8(29.63)	19(70.37)	27	0.94(0.72-1.24)	0.678
III	2(15.38)	11(84.62)	13	1.14(0.88-1.47)	0.334
Total	36(25.35)	106(74.65)	142		
Severity of burns (ABA)					
Mild	3(60.00)	2(40.00)	5	1(ref)	
Moderate	11(29.73)	26(70.27)	37	1.76(0.59-5.24)	0.313
Severe	22(22.00)	78(78.00)	100	1.95(0.66-5.73)	0.225
Total	36(25.35)	106(74.65)	142		
Facial/inhalational burns					
Facial or inhalational	14(26.42)	39(73.58)	53	1.05(0.84-1.31)	0.66
Both	1(5.26)	18(94.74)	19	1.35(1.12-1.63)	0.001
None	21(30.00)	49(70.00)	70	1(ref)	
Total	36(25.35)	106(74.65)	142		
First aid time delay					
≤1 hour	10(20.83)	38(79.17)	48	1.09(0.90-1.33)	0.356
>1 hour	26(27.66)	68(72.34)	94	1(ref)	
Total	36(25.35)	106(74.65)	142		
Pre-hospital measures					
Yes	3(21.43)	11(78.57)	14	1.05(0.79-1.41)	0.702
No	33(25.78)	95(74.22)	128	1(ref)	
Total	36(25.35)	106(74.65)	142		

In adjusted analysis, we found that female gender (adjusted prevalence ratio: 1.33 (95% CI 1.17-1.52; p<0.01) and patients having both facial and inhalational burns (adjusted prevalence ratio: 1.27 (95% CI 1.16-1.40; p<0.01) were found to be independently associated with a longer hospital stay (Table 4).

**Table 4:** Multivariate logistic regression for factors associated with duration of hospital stay among admitted alive study participants in EMD of a tertiary care center N=142 aPR, adjusted prevalence ratio; CI, confidence interval; EMD, Emergency Medicine Department; TBSA, total body surface area Severity of burns classification - refer to appendices (Table 6)

Characteristics	Total (%)	>1 week of hospital stay	aPR (95% CI)	P-value
Gender				
Male	88(61.97)	61(69.32)	1(ref)	
Female	54(38.03)	45(83.33)	1.33(1.17-1.52)	<0.001
Burns TBSA (%)				
<10	11(7.75)	6(54.55)	1(ref)	
10-30	102(71.83)	75(73.53)	1.18(0.69-2.04)	0.545
31-50	29(20.42)	25(86.21)	1.05(0.61-1.81)	0.863
Severity of burns				
Mild	5(3.52)	2(40.00)	1(ref)	
Moderate	37(26.06)	26(70.27)	1.28(0.44-3.67)	0.650
Severe	100(70.42)	78(78.00)	1.55(0.54-4.46)	0.413
Facial/inhalational burns				
Facial or inhalational	53(37.32)	39(73.58)	0.89(0.73-1.07)	0.221
Both	19(13.38)	18(94.74)	1.27(1.16-1.40)	<0.001
None	70(49.30)	49(70.00)	1(ref)	

The percentage of burns, mode of injury, and severity of burns were found as significant predictors of length of hospital stay from linear regression analysis. The simple linear regression analysis for predictors of length of hospital stay is shown in Table 5.

**Table 5:** Simple linear regression for factors associated with duration of hospital stay among admitted study participants in EMD of a tertiary care center N=142 CI, confidence interval; EMD, Emergency Medicine Department Severity of burns classification - refer to appendices (Table 6)

Variable	Constant	β-coefficient	95% CI		P-value
			Lower limit	Upper limit	
Gender (male/female)	12.08	2.49	-1.75	6.73	0.249
Percentage of burns	7.51	0.34	0.17	0.52	<0.001
Mechanism of burns (thermal/electrical/chemical)	13.32	1.71	-0.84	4.26	0.187
Mode of burn injury (suicidal/homicidal/accidental)	30.88	-5.43	-9.13	-1.73	0.004
Degree of burns (I/IIs/IId/III)	11.35	3.04	-0.13	6.20	0.060
Severity of burns (mild/moderate/severe)	2.75	4.78	1.03	8.53	0.013
Facial/inhalational burns (Yes/No)	15.19	0.15	-2.09	2.39	0.894
Pre-hospital measures (Yes/No)	13.01	2.28	-4.65	9.22	0.516

## Discussion

Our study adopted a cross-sectional analytical design to assess the proportion of burn patients having longer hospital stays (more than one week) and the factors associated with it. It was done among patients with burn injuries, who attended the EMD of a tertiary health facility in south India. The proportion of live admitted patients having a longer duration of stay was found to be 74.6% (95% CI 66.8-81.2). Gender and facial/inhalational burns were found to have an independent effect on the duration of hospital stay. The case fatality rate observed during the study was 35.8% (N=117).

National Programme for Prevention and Management of Burn Injuries (NPPMBI)

There has always been a lack of authentic national data on burns succored by non-existent central registries maintained for burns cases. This paucity of data is compounded by the absence of management protocols, referral mechanisms, dedicated burn units, and trained manpower not only among the primary and secondary care level but also among the private sector. One such important reason for these unreliable estimates could be due to the fact that burns are often not notified or recorded, although it mandates a medico-legal registration. Considering the magnitude of this issue, the Ministry of Health and Family Welfare (MoHFW) and Directorate General of Health Services have implemented NPPMBI, functional since 2014 under the twelfth five-year plan, to strengthen the preventive, curative, and rehabilitative services for burn victims. Since its inception, the emphasis had been laid through several strategies like media engagement, capacity building, need-based behavior change communication, awareness generations, referral linkage, data management, training manpower, and setting up dedicated burns care units across varied primary, secondary, and tertiary care settings.

Length of hospital stay

Among patients discharged from the hospital (142), 75% of them had a longer duration of hospital stay. Similar results were observed in studies done in various other parts of the country [5]. Research from outside India has also found similar proportions, especially in a tertiary care setting [6]. We found the female gender to be an independent factor in determining hospital stay; this finding is also supported by other studies [7-11]. Facial/inhalational burn injuries were also found to be independently associated with the length of hospital stay, which in turn is proved by research from other study settings [10]. As we considered for analysis, the duration of hospital stay might not always correlate positively (while we expect a negative co-relation) with the severity of burns or TBSA of burns, as even a moderate degree of burns, if proceeds into complications such as sepsis, can prolong the duration of hospital stay. The duration of hospital stay is expected to be less among severe burns and patients with >50% TBSA of burns as they succumb to death early [12]. Even in our study, we found that the vast majority with severe burns or greater TBSA% died early within one week, whereas mild-to-moderate burns had longer hospital stays as they needed better care. Though this need not be always true in every study setting, as it is also influenced by other factors like delay in care, type of health center, and availability of services. Facial and inhalational burns are expected to have a poorer outcome as it leads to the development of acute respiratory distress syndrome (ARDS) and, in turn, respiratory dysfunction, thereby warranting a longer hospital stay or intensive care unit care. This type of injury, which is commonly encountered, also mandates the requirement of advanced resuscitative measures in the immediate phase, and reconstructive surgery in the late phase of rehabilitation, thereby highlighting the importance of dedicated burns units in a tertiary care setting. 

Clinico-demographic profile

We also found that the percentage of burns, mechanism of burns, and pre-hospital measures were found to be statistically significant predictors of the length of hospital stay [13-15]. Similar results were obtained from previous studies published in different parts of the world [13-15]. We also observed that burn injuries were more common among women and children, taking place mostly at home and accidental by means. The observation of female predominance among burn patients was in line with other studies from similar settings in south India [5,16,17]. This cannot be frankly extrapolated to the female gender only because they do more household chores such as open-fire cooking practices or inherently using unsafe cookstoves, which can ignite loose clothing. Women being more prone to emotional disturbances and interpersonal violence might also inflate their risk. This fact also highlights the underpinning of social and phycological factors prevailing in our society that largely affect and increase the susceptibility of such vulnerable populations. Accidental injuries, the most commonly encountered mode of injury in our study, also target the female population who are readily available in the kitchen most of the time. This further emphasizes the necessity to inculcate safety measures such as guarding electrical sockets, covering hot utensils, and keeping the fireplace out of reach of children. Several studies have highlighted the need for better safety provisions at home, thereby preventing the majority of burns.

The most common mode of burns injury encountered was accidental (74%), which was on par with other research articles [4,18-22]. This could probably reflect the prevailing lifestyle practices, improper kitchen design, unsafe cooking practices, and lack of home safety, among others. As in any emergency health care, pre-hospital response plays an important role in mitigating an injury by providing immediate life-saving services. In the present study, only 14 (10%) patients had received pre-hospital measures before presenting to the health center. But it was noted that all of them received inappropriate pre-hospital measures like a banana leaf, ink, and paste application over burn areas, which increases the chance of sepsis and mortality. Further studies are required to gain a better understanding of the role of these indigenous practices. Almost two-thirds of the patients who visited the center had a time delay of more than one hour. This, in turn, emphasizes the need to strengthen transportation services and operationalize dedicated centers at the sub-district level. The existing emergency 108 services need to be further strengthened to bridge this gap.

Limitations

Our study had a few limitations. First, being a single-center study from southern India, the results may not be generalizable to other hospitals from different settings. Second, being an institution-based study, it may not reflect the true burden of the disease. Third, we did not account for the treatment outcome in the analysis as it was not our primary objective. Fourth, we did not account for the associated co-morbidity or complications developed during the treatment for ascertaining the length of hospital stay. We recommend further research to explore this aspect. Despite these pitfalls, we have compared the length of hospital stay with an exhaustive list of clinico-demographic determinants supported by subgroup analysis only among the admitted alive patients to determine the length of hospital stay, thereby minimizing the risk of misclassification bias.

## Conclusions

We observed that of the admitted alive burn injury patients, around three fourth had a longer duration of stay (more than one week). Female gender and facial/inhalational burns stood out as independent factors determining the length of hospital stay. Thus, through this study, we identify the need for better home safety, childproofing, necessary education, proper pre-hospital care, and dedicated burns units with trained manpower for better care and safety of individuals. Burn injuries, being preventable by large, needs to be tackled by strict laws enforcing environmental safety at homes and workplace. This, if implemented in a proper way, would definitely minimize the economic and social burden levied on the tertiary health care system.

## Appendices

**Table 6:** Severity of burns based on the ABA A combination of the burn mechanism, burn depth, extent, and anatomic location determines the overall severity of the burn injury, which provides general guidance for the preferred disposition and care of these patients. Minor or mild burn injury: minor or mild burns are those that can be treated in a physician's office or in an emergency department as an outpatient, moderate burn injury: moderate burns would be those that require admission to a hospital but not to a burn center. These include superficial burns or deeper burns of a limited extent. Severe burn injury: severe burn injury can be defined as burns that should be referred to, and treated at, a designated burn center. ABA, American Burns Association

	Mild		Moderate	Severe
Children				
Partial thickness burn	<10% BSA		10-20% BSA	>20% BSA
Full thickness burn	<2% BSA		2-10% BSA	>10% BSA
Adults				
Partial thickness burn	<15% BSA		15-25% BSA	>25% BSA
Full thickness burn	<2% BSA		2-10% BSA	>10% BSA
Age			Patients <2 years with a minor injury	Patients <10 years with major injury
Hands, face, feet, perineum - burns		(-)	(-)	Moderate injury involvement
Electrical injury	(-)		(-)	(+)
Chemical injury	(-)		(-)	(+)
Inhalational Injury	Not suspected		Not suspected	(+)
Major associated medical illness	(-)		(-)	(+)
Associated fractures, multiple trauma	(-)		(-)	(+)
